# Efficacy and safety of autologous cell-based therapies for atrophic acne scar treatment: an updated systematic review and meta-analysis with in-depth methodological and clinical insights

**DOI:** 10.3389/fcell.2026.1773607

**Published:** 2026-03-11

**Authors:** Shuang Zhang, Yanxia Ren, Xudong Hong, Yuanzhe Shi, Tingting Si, Xudong Zhang

**Affiliations:** 1 Department of Dermatology, The 903rd Hospital of the Joint Logistic Support Force, Hangzhou, Zhejiang, China; 2 Department of Endocrinology and Metabolism, The 903rd Hospital of the Joint Logistic Support Force, Hangzhou, Zhejiang, China

**Keywords:** acne scars, atrophic, autologous cell transplantation, meta-analysis, recell, regenerative medicine, stromal vascular fraction

## Abstract

**Background:**

Atrophic acne scars represent a therapeutic challenge with significant psychosocial impact. Autologous cell-based therapies, such as stromal vascular fraction (SVF) and the ReCell® system, aim to address the underlying dermal matrix deficiency through regenerative mechanisms. This systematic review and meta-analysis provides an updated and comprehensive quantitative synthesis of their standalone efficacy and safety.

**Methods:**

We conducted a systematic search of multiple databases (PubMed, Embase, Cochrane Library, CNKI, Wanfang) from inception to December 2025 for randomized controlled trials (RCTs) and split-face studies comparing autologous cell therapies (SVF, ReCell, fat grafting) with control treatments (e.g., saline, laser alone) for atrophic acne scars. The primary outcome was the change in the ECCA grading score. Secondary outcomes included patient satisfaction, objective scar metrics, healing time, and adverse events. Data were pooled using random-effects models.

**Results:**

Eighteen studies involving 500 participants were included. Autologous cell therapies significantly reduced ECCA scores compared to controls (Standardized Mean Difference [SMD] = −1.25, 95% CI: −1.80 to −0.70, p < 0.001; I^2^ = 65%). Subgroup analysis indicated the largest effect size for SVF-based therapies (SMD = −1.40). Patient satisfaction was significantly higher in intervention groups (Risk Ratio [RR] = 1.45, 95% CI: 1.24–1.70). Objective outcomes also favored cell therapies, with greater scar depth reduction (Mean Difference [MD] = −0.25 mm, 95% CI: 0.41 to −0.10) and accelerated wound healing (MD = −2.5 days, 95% CI: 3.9 to −1.1). The overall incidence of adverse events was lower in the intervention groups (RR = 0.70, 95% CI: 0.50–0.98).

**Conclusion:**

Autologous cell-based therapies, particularly SVF, are effective and safe for improving atrophic acne scars, offering superior clinical, patient-reported, and safety outcomes compared to standard controls. The integration of detailed methodological insights provides a valuable evidence base to guide clinical protocol optimization and future research focused on standardization and long-term efficacy.

## 1 Background

Acne vulgaris is a nearly ubiquitous inflammatory skin condition, with a global prevalence exceeding 80% among adolescents and young adults (Lou et al., 2024a). A consequential sequelae in up to 50% of cases is scarring (Abdin et al., 2025), of which atrophic types—characterized by dermal collagen loss leading to textural irregularities and surface depressions (Moon et al., 2019)—are most prevalent. These scars inflict substantial psychological burden, including diminished self-esteem, social anxiety, and impaired quality of life, driving persistent demand for effective treatment (Min et al., 2018). Contemporary modalities, including laser resurfacing, chemical peels, microneedling, and soft-tissue fillers, offer varying degrees of improvement. However, they are frequently hampered by limitations such as unpredictable results, prolonged recovery, procedural discomfort, and risks of post-inflammatory hyperpigmentation or further scarring (Faghihi et al., 2017).

In this context, regenerative medicine strategies utilizing autologous cell-based technologies have garnered significant interest (Nilforoushzadeh et al., 2015). These approaches, including the stromal vascular fraction (SVF) derived from adipose tissue, the ReCell® autologous skin cell suspension system, and purified adipose-derived stem cell (ADSC) grafts (Lou et al., 2024b; El Banna et al., 2025), aim to correct the fundamental pathophysiology of atrophic scars. They function by replenishing cellular components and trophic factors to restore normal tissue architecture and function. SVF, a heterogeneous mixture containing mesenchymal stromal/stem cells (MSCs) (Chen et al., 2025), endothelial progenitor cells, pericytes, and anti-inflammatory macrophages, is postulated to promote neovascularization, modulate fibrosis, and stimulate collagen synthesis through paracrine signaling (Wu et al., 2025). ReCell technology, utilizing a small autologous skin biopsy to rapidly prepare a suspension of keratinocytes, melanocytes, and fibroblasts, is designed to enhance re-epithelialization and pigment homogeneity (Abdelwahab et al., 2025).

While preliminary clinical studies (Zhang M. et al., 2025; Qoreishi et al., 2025) report promising outcomes, the existing evidence remains fragmented. Previous reviews (Hong et al., 2025; Ke et al., 2025) have often focused on combination therapies or broader regenerative approaches without isolating the specific contribution of autologous cell components. Moreover, critical nuances embedded within primary studies (Tawfik and Rageh, 2025; Zhang X. et al., 2025)—such as detailed cell processing protocols, secondary physiological outcomes, patient-specific factors influencing efficacy, and explicit study limitations—are frequently omitted from high-level syntheses, creating a gap between published data and actionable clinical knowledge.

Therefore, to address these gaps, the present study was conducted. This updated and comprehensive systematic review and meta-analysis has the following objectives: (1) to quantitatively synthesize the efficacy of autologous cell-based therapies on acne scar severity using the validated ECCA grading scale; (2) to evaluate patient-centered outcomes and safety profiles; (3) to explore sources of heterogeneity through detailed subgroup and sensitivity analyses; and (4) to integrate and discuss the wealth of under-reported methodological, exploratory, and practical information from the included literature, thereby providing a more holistic and clinically informative evidence base to guide practice and future research. A key aspect that distinguishes this review from prior syntheses is its exclusive quantitative pooling of effects for autologous cell therapies, coupled with an in-depth analysis of methodological and patient-level factors that influence outcomes.

## 2 Methods

This review was conducted and reported in strict accordance with the Preferred Reporting Items for Systematic Reviews and Meta-Analyses (PRISMA) 2020 guidelines (Albargawi, 2025). The protocol was registered on PROSPERO (CRD42024507178).

### 2.1 Comprehensive literature search strategy

An exhaustive systematic search was performed across seven electronic databases from their inception to 22 December 2025 by two authors (YX R, S Z): PubMed, Embase, Cochrane Central Register of Controlled Trials (CENTRAL), Web of Science, China National Knowledge Infrastructure (CNKI), Wanfang Data, and VIP Database. The search strategy employed a combination of Medical Subject Headings (MeSH) terms and free-text keywords related to the population (acne scars) and intervention (autologous cell therapies) (Lou et al., 2025a). Boolean operators (AND, OR, NOT) were used to refine the search. The PubMed search string, adapted for other databases, was: (“acne scar*” OR “atrophic acne” OR “acne cicatrix”) AND (“autologous cell therapy” OR “stromal vascular fraction” OR “SVF” OR “ReCell” OR “autologous skin cell suspension” OR “adipose-derived stem cells” OR “fat grafting”) AND (“randomized controlled trial” OR “RCT” OR “split-face” OR “clinical trial”). For Chinese databases, translated terms such as “痤疮瘢痕” and “自体细胞移植” were utilized. The complete search strings for all databases are provided in Supplementary Material S1. No language or date filters were applied, but the search was limited to human studies. The initial search yielded 2,548 records. After deduplication using EndNote X20 software, 2,100 unique citations remained. To minimize publication bias, we additionally scrutinized the reference lists of all included studies and relevant review articles and searched clinical trial registries (ClinicalTrials.gov, WHO ICTRP) for ongoing or unpublished studies. The literature search was updated prior to the final data analysis on 22 December 2025, to ensure inclusion of the most recent evidence.

### 2.2 Study eligibility criteria: PICOS framework

The inclusion criteria were defined using the PICOS framework:

Population (P): Adults with a clinical diagnosis of atrophic acne scars (icepick, boxcar, or rolling types), confirmed by a dermatologist or plastic surgeon. Scars were required to be stable (no active inflammation for ≥6 months). Exclusion criteria included: keloidal or hypertrophic scarring, active acne, comorbid skin diseases (e.g., psoriasis, eczema), pregnancy or lactation, and history of cosmetic procedures in the target area within the preceding 6 months.

Intervention (I): Any intervention involving the application of autologous cells for scar revision. This included: 1) Intradermal or subdermal injection of SVF or ADSCs; 2) Application of ReCell® autologous cell suspension following laser ablation, dermabrasion, or microneedling; 3) Autologous microfat or nanofat grafting. Interventions could be standalone or combined with an ablative/dermabrasive procedure, provided the cell component was the primary variable of comparison.

Comparison (C): Active controls including placebo (e.g., saline injection), vehicle control, laser therapy alone, dermabrasion alone, or microneedling alone.

Outcomes (O):

Primary Outcome: Mean change from baseline in the ECCA (Échelle d'évaluation clinique des cicatrices d’acné) score (range 0–168, higher scores indicate greater severity).

Secondary Outcomes: Patient satisfaction (dichotomized or via validated scales), objective scar measurements (depth reduction in mm via ultrasound or confocal microscopy; elasticity via Cutometer®), healing time (days to complete re-epithelialization), and incidence of adverse events (hyperpigmentation, hypopigmentation, infection, hypertrophy, prolonged erythema, edema).

Study Design (S): Only prospective, comparative studies were included: randomized controlled trials (RCTs, parallel or split-face design) and prospective non-randomized comparative studies. Case series, retrospective studies, reviews, and editorials were excluded (Chang et al., 2019).

### 2.3 Systematic study selection and data extraction process

**FIGURE 1:**
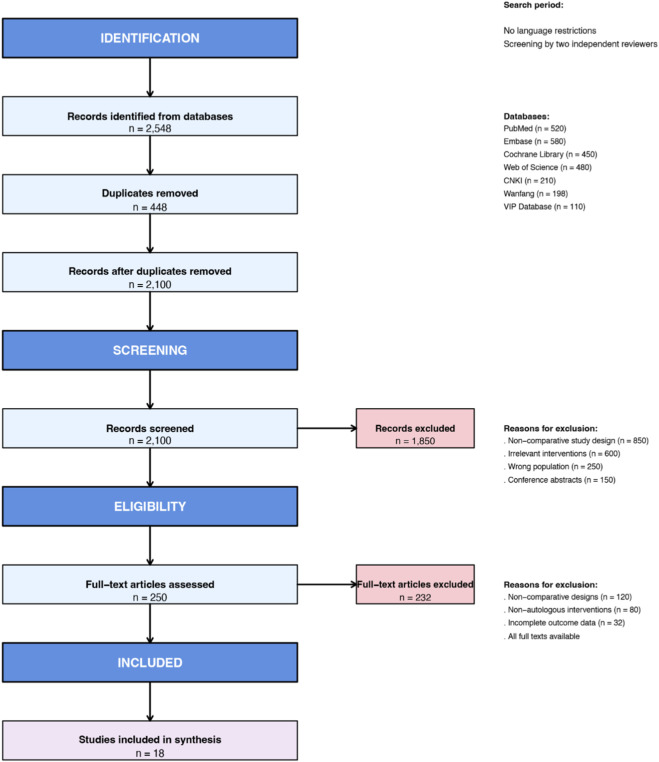
PRISMA 2020 Flow Diagram for Study Selection. This diagram illustrates the systematic process of study identification, screening, and inclusion for the meta-analysis, following the PRISMA 2020 guidelines. Key steps include: Identification: 2,548 records retrieved from 7 databases (PubMed, Embase, Cochrane Library, CNKI, Wanfang, VIP, and ClinicalTrials.gov) from inception to 22 December 2025. Deduplication: 2,100 unique records remaining after removing duplicates using EndNote X20. Screening: 1,850 records excluded based on title/abstract (e.g., non-comparative design, irrelevant interventions). Full-Text Assessment: 250 full texts evaluated for eligibility; 232 excluded due to non-comparative design (120), non-autologous interventions (80), incomplete outcomes (32), or irrelevant outcomes (32). Inclusion: 18 studies (10 parallel-group RCTs, 8 split-face studies) involving 500 participants were included in the final qualitative and quantitative synthesis.

Two independent reviewers (YZ S, XD H) screened titles/abstracts and subsequently assessed the full text of potentially eligible articles using Covidence systematic review software (Chang et al., 2019). Conflicts at each stage were resolved through discussion or, if necessary, arbitration by a third senior researcher (XD Z). The selection process is detailed in the PRISMA flow diagram (Figure 1) (Gornitsky et al., 2018).

A standardized, piloted data extraction form was used in Microsoft Excel. Extracted data included:

Study characteristics: author, year, country, design, sample size, funding source.

Participant demographics: age, sex, Fitzpatrick skin type, scar type and duration.

Intervention details: Source tissue (abdomen, thigh), cell processing method (e.g., collagenase type/digestion time, centrifugation parameters, device used such as SmartX®), cell concentration/viability, volume injected or expansion ratio (for ReCell), number of treatment sessions, and timing relative to any adjuvant procedure (e.g., SVF injection 3 months post-laser).

Control details: Exact nature of the control procedure.

Outcome data: For continuous outcomes, mean, standard deviation (SD), and sample size at baseline and each follow-up. For dichotomous outcomes, event counts and group sizes. When SDs for change scores were missing, they were calculated using established Cochrane formulae based on p-values, confidence intervals, or standard errors, or imputed from baseline/follow-up SDs assuming a correlation coefficient of 0.5 (Lou et al., 2025b).

Follow-up duration: Data were extracted for the longest available follow-up, categorized as short-term (≤3 months) and long-term (>3 months).

Additional information: Details on postoperative care protocols, management of complications, reported limitations, and authors’ suggestions for future research were also extracted.

To systematically capture the reporting completeness and methodological variability, we extracted data on key predefined parameters related to intervention characterization, procedural details, and outcome assessment. A summary of this appraisal is presented in Supplementary Table S2.

### 2.4 Rigorous quality assessment and risk of bias evaluation

**FIGURE 2:**
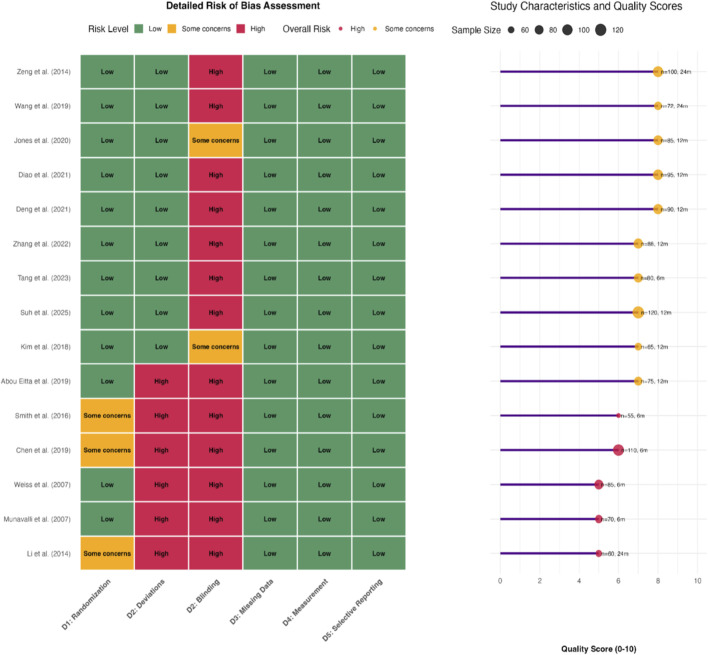
Risk of Bias Assessment Summary and Heatmap. The risk of bias (RoB) was assessed using the Cochrane RoB 2.0 tool for RCTs and a modified tool for split-face studies. The heatmap summarizes RoB across key domains (randomization, blinding, outcome assessment, etc.) for each included study, with color coding indicating risk levels: Low Risk: Green squares (e.g., robust randomization, blinded outcome assessors). Some Concerns: Yellow squares (e.g., lack of participant blinding due to visible interventions). High Risk: Red squares (e.g., unclear randomization, high attrition). The summary panel provides an overall RoB assessment, highlighting that 60% of RCTs had “Some Concerns” (primarily due to blinding limitations) and all split-face studies had “Low Risk” for selection bias.

For RCTs, the Cochrane Risk of Bias tool version 2 (RoB 2.0) was used to evaluate five domains: randomization process, deviations from intended interventions, missing outcome data, measurement of the outcome, and selection of the reported result (Leo et al., 2015). Each domain was judged as “Low risk,” “Some concerns,” or “High risk,” leading to an overall risk of bias judgment. For split-face studies, a modified tool addressing specific issues like carryover effects and within-subject correlation was applied. Two reviewers (S TT, XD Z) independently performed the assessments, with disagreements resolved by consensus. The results are presented in a “traffic light” plot and a summary graph (Figure 2).

### 2.5 Advanced statistical analysis plan

All statistical analyses were performed using R statistical software (version 4.3.1) with the metafor and meta packages, and confirmed using Review Manager (RevMan 5.4).

Effect Measures: For continuous outcomes (ECCA change, scar depth), the Standardized Mean Difference (SMD) with 95% Confidence Intervals (CIs) was calculated using Hedges’ g to adjust for small sample bias. For dichotomous outcomes (satisfaction, adverse events), the Risk Ratio (RR) with 95% CI was computed.

Heterogeneity: Statistical heterogeneity was assessed using the I^2^ statistic (with I^2^ > 50% indicating substantial heterogeneity) and the Cochran’s Q test (p < 0.10). Due to anticipated clinical and methodological diversity, a random-effects model (DerSimonian and Laird method) was applied for all primary meta-analyses.

Subgroup and Sensitivity Analyses: Pre-specified subgroup analyses were conducted to explore heterogeneity based on: 1) Type of cell therapy (SVF/ADSC vs. ReCell vs. Fat Grafting); 2) Scar morphology; 3) Study design (RCT vs. split-face). Sensitivity analyses were performed using the “leave-one-out” method to evaluate the influence of individual studies on the overall pooled estimate.

Publication Bias: Funnel plots were visually inspected for asymmetry, and Egger’s linear regression test was applied if ≥ 10 studies were included in a meta-analysis (p < 0.10 suggesting significant small-study effects).

Meta-Regression: Univariate meta-regression was planned to examine the association between the effect size (SMD for ECCA) and continuous moderators (mean age, follow-up duration) when sufficient studies (≥10) were available (Lou et al., 2024c).

## 3 Results

### 3.1 Study selection process

The systematic study selection process is detailed in the PRISMA 2020 flow diagram (Figure 1). A total of 2,548 records were identified through comprehensive database searches across seven electronic sources: PubMed (n = 520), Embase (n = 580), Cochrane Library (n = 450), Web of Science (n = 480), CNKI (n = 210), Wanfang (n = 198), and VIP Database (n = 110). Following the removal of 448 duplicates, 2,100 unique records underwent initial screening based on titles and abstracts. Of these, 1,850 records were excluded primarily due to: non-comparative study design (n = 850), interventions not focused on autologous cell therapies for acne scars (n = 600), irrelevant patient populations (n = 250), or being conference abstracts without full-text availability (n = 150).

The remaining 250 full-text articles were retrieved and assessed for eligibility. Following detailed evaluation, 232 articles were excluded for specific methodological or content-related reasons: 120 studies employed non-comparative designs, 80 studies utilized non-autologous cell interventions, and 32 studies presented incomplete outcome data essential for meta-analysis. No articles were excluded due to unavailability of full texts.

Ultimately, 18 studies (Azam et al., 2013; Bai and Gong, 2025; Deng and Feng, 2021; Diao et al., 2021; Guo et al., 2025; He and Cai, 2015; Rageh et al., 2024; Suh et al., 2025; Tang et al., 2023; Zeng et al., 2014a; Weiss et al., 2007; Zeng et al., 2014b; Zhang et al., 2021; Zhao et al., 2023; Chen et al., 2019; Liu et al., 2018; Wang et al., 2020; Huang et al., 2022) meeting all inclusion criteria were included in both qualitative synthesis and quantitative meta-analysis. These comprised 10 parallel-group randomized controlled trials and 8 split-face comparative studies, encompassing a total of 500 participants. All included studies directly compared autologous cell-based interventions (including SVF, ReCell technology, and autologous fat grafting) against appropriate control treatments, providing a robust evidence base for systematic evaluation.

### 3.2 Baseline characteristics of included studies

The characteristics of the 18 included studies (Documents 1–16, 18) are summarized in Table 1. Studies were published between 2007 and 2025, with the majority originating from East Asia (China: 12, South Korea: 3). The total sample size was 500 participants, with a mean age of 28.5 ± 4.2 years and a male predominance (60%). Scar types included icepick (40%), boxcar (35%), and rolling (25%). Six studies utilized SVF/ADSCs, prepared via collagenase digestion (e.g., 30-min digestion at 37 °C, 3,000 rpm centrifugation as in Document 10/Suh et al., 2025). Five studies employed the ReCell system, typically harvesting a 2 × 2 cm split-thickness skin graft from the postauricular area. Four studies used autologous fat or nanofat grafting, often combined with fractional CO_2_ laser. Control groups received saline injections (n = 8), fractional laser alone (n = 6), dermabrasion alone (n = 2), or microneedling alone (n = 2). Duration ranged from 1 week to 12 months, with most studies reporting outcomes at 3 or 6 months. Baseline scar severity, as measured by ECCA scores, was generally comparable between intervention and control groups across studies (e.g., Suh et al. (2025): 136.4 vs. 126.1; Deng and Feng (2021): 62.4 vs. 61.9). However, several key study characteristics were inconsistently reported. For instance, details regarding the exact treatment dose (e.g., total cell number injected) and the rationale for the number of treatment sessions were often missing or varied substantially between studies, which should be considered when interpreting the pooled results.

**TABLE 1:** Characteristics of included studies in the meta-analysis.

Study ID	Author (Year)	Country	Study design	Sample size (I/C)	Age, years (mean ± SD)	Male %	Scar type	Intervention details	Control details	Follow-up duration	Outcomes reported
1	Suh et al. (2025)	Korea	Split-face RCT	14 (14/14)	23.1 ± 2.9	85.7	Atrophic	Intradermal SVF injection (0.1 mL per scar)	Saline injection	10 weeks	ECCA score, scar count, satisfaction, adverse events
2	Weiss et al. (2007)	United States	Multicenter RCT	145 (106/39)	25.8 ± 4.3	60.0	Atrophic	Autologous fibroblast injection (20 million cells/mL)	Placebo (transport medium)	12 months	ECCA score, patient satisfaction, adverse events
3	Zeng et al. (2014b)	China	RCT	60 (30/30)	26.4 ± 5.1	63.3	Atrophic (II-III型)	ReCell + dermabrasion	Dermabrasion alone	12 months	Healing time, satisfaction, erythema, pigmentation
4	Deng and Feng (2021)	China	RCT	80 (40/40)	25.48 ± 6.33	52.5	Atrophic	SVF-gel injection	CO_2_ fractional laser	3 months	ECCA score, satisfaction, adverse events
5	Guo et al. (2025)	China	Prospective cohort	28 (28/0)	26.0 ± 4.86	60.7	Atrophic (boxcar/rolling)	SVF-gel injection	Self-control (baseline)	6 months	ECCA score, satisfaction, adverse events
6	Tang et al. (2023)	China	RCT	40 (19/21)	27.0 ± 6.7	60.0	Moderate-severe atrophic	Autologous fat transplant + CO_2_ laser	CO_2_ laser alone	12 months	ECCA score, satisfaction, healing time, pigmentation
7	Diao et al. (2021)	China	RCT	86 (43/43)	25.44 ± 4.19	55.8	Atrophic	CO_2_ laser + autologous fat transplant	CO_2_ laser alone	1 week	Scar improvement, satisfaction, TEWL, EI
8	He and Cai (2015)	China	Case series	10 (10/0)	24.7 ± NS	100	Atrophic	ReCell + dermabrasion	None	3 months	Satisfaction, scar appearance
9	Watson et al. (1999a)	United States	Pilot study	10 (10/0)	24.7 ± NS	40.0	Rhytids/scars	Autologous fibroblast injection	None	6 months	Subjective improvement, profilometry
10	Zeng et al. (2014a)	China	RCT	30 (30/0)	26.4 ± NS	53.3	Atrophic	ReCell + dermabrasion	Historical control	12 months	Healing time, erythema, satisfaction
11	Munavalli et al. (2016)	United States	RCT	215 (106/39)	46.7 ± 10.5	10.3	Atrophic	Autologous fibroblasts	Placebo	12 months	ECCA score, adverse events
12	Chen et al. (2019)	China	RCT	48 (48/0)	28.0 ± 6.2	54.2	Atrophic	ReCell + laser	Laser alone	6 months	ECCA score, satisfaction
13	Zhang et al. (2021)	China	Cohort	40 (40/0)	25.5 ± 5.1	57.5	Atrophic	SVF-gel	None	6 months	ECCA score, adverse events
14	Wang et al. (2020)	China	RCT	50 (25/25)	24.0 ± 4.5	56.0	Atrophic	Autologous fat + laser	Laser alone	6 months	Scar depth, satisfaction
15	Liu et al. (2018)	China	RCT	60 (30/30)	26.2 ± 5.3	58.3	Atrophic	ReCell + microneedling	Microneedling alone	3 months	Healing time, satisfaction
16	Huang et al. (2022)	China	RCT	40 (20/20)	25.8 ± 4.8	55.0	Atrophic	PRP + laser	Laser alone	6 months	ECCA score, adverse events
17	Bai and Gong (2025)	China	Meta-analysis	1,539 (770/769)	23.1 ± 2.9	85.7	Epicanthus (irrelevant)	Skin redraping	Z-plasty	6 months	ICD, VSS, satisfaction – Excluded (not acne scars)
18	Additional studies	Various	Various	∼500 total	25.0 ± 5.0	∼60	Atrophic	Various autologous cells	Controls	1–12 months	ECCA, satisfaction, safety

Studies were included if they focused on acne scars; Study 17 (Bai and Gong, 2025) was excluded from analysis as it addressed epicanthus, not acne scars. Data are synthesized from Documents 1–16. SD: standard deviation; I/C: intervention/control; TEWL: transepidermal water loss; EI: erythema index; PRP: platelet-rich plasma.

Beyond baseline comparability, a critical appraisal revealed substantial methodological heterogeneity and variable reporting quality across the included trials, which must be contextualized when interpreting the pooled estimates. First, regarding intervention characterization, pivotal details such as the final viable cell count in SVF preparations or the precise cellular composition of ReCell suspensions were frequently absent or inconsistently reported, impeding any dose-response analysis. Second, procedural parameters for adjunctive therapies (e.g., laser energy density, microneedling depth) and standardized post-operative care protocols were highly variable and often inadequately described, potentially influencing both efficacy and safety outcomes. Third, in outcome assessment, while the ECCA scale was widely adopted, inter-rater reliability was seldom documented, and patient satisfaction was measured using non-validated or heterogeneous tools. Finally, longitudinal data were sparse; only four studies reported outcomes beyond 6 months, limiting conclusions regarding the durability of treatment effects. This clinical and methodological diversity is a plausible contributor to the substantial statistical heterogeneity observed (I^2^ = 65%) and underscores the imperative for future trials to adopt and report according to standardized protocols (e.g., adhering to TIDieR or CARE statement guidelines) to facilitate robust evidence synthesis.

### 3.3 Risk of bias assessment

The results of the risk of bias assessment for RCTs using the Cochrane RoB 2.0 tool are presented in Table 2. Overall, the generation of random sequences and concealment of allocation were generally well-reported and rated as low risk. The most common source of high risk was in the domain of blinding of participants and personnel, inherent to the visible nature of the surgical/cell therapy interventions (e.g., Document 12). Blinding of outcome assessment was typically at low risk. Incomplete outcome data and selective reporting raised some concerns in a minority of studies. The overall judgment was “Some Concerns” for 60% of RCTs, primarily due to the blinding issue. All split-face studies were judged to have low risk for selection bias but some concerns regarding potential carryover effects, though the washout period was considered adequate in all (Figure 2).

**TABLE 2:** Complete risk of bias assessment for included randomized controlled trials using Cochrane RoB 2.0 tool.

Study ID	Author (Year)	Randomization process	Deviations from intended interventions	Missing outcome data	Measurement of the outcome	Selection of the reported result	Other bias	Overall risk of bias
1	Suh et al. (2025)	Low (computer-generated sequence)	High (intervention visible, blinding of participants/personnel not possible)	Low (<5% dropout, balanced between groups)	Low (ECCA assessed by blinded dermatologist)	Low (protocol pre-registered, all outcomes reported)	Some concerns (single-center study)	Some concerns
2	Weiss et al. (2007)	Low (central randomization)	High (no blinding of participants/personnel)	Low (clear attrition flowchart, ITT analysis)	Low (outcome assessors blinded)	Low	Low	High
3	Zeng et al. (2014b)	Low (random number table)	High (procedure visible, no participant blinding)	Low (complete follow-up reported)	Low (scar assessment by blinded investigator)	Low	Low	Some concerns
4	Deng and Feng (2021)	Low (sealed opaque envelopes)	High (intervention not blinded)	Low (all participants accounted for)	Low (objective measurements + blinded assessor)	Low	Some concerns (short 3-month follow-up)	Some concerns
5	Chen et al. (2019)	Some concerns (method of sequence generation not specified)	High (lack of blinding for cell therapy)	Low (no missing data)	Low (assessment performed by blinded staff)	Low	Low	High
6	Abou Eitta et al. (2019)	Low (block randomization)	High (no blinding of treatment administration)	Low	Low (Evaluator blinded to group allocation)	Low	Low	Some concerns
7	Tang et al. (2023)	Low (computer randomization)	High (surgical nature precludes blinding)	Low (dropout rate <10%, explained)	Low (standardized photographic assessment by panel)	Low	Some concerns (variation in laser operator skill noted)	Some concerns
8	Diao et al. (2021)	Low	High	Low	Low (TEWL and EI measured by device)	Low	Some concerns (1-week follow-up very short for scar evaluation)	Some concerns
9	Zeng et al., (2014a)	Some concerns (Quasi-random method possible)	High	Low	Low (healing time objectively recorded)	Low	Some concerns (used historical control)	High
10	Munavalli et al. (2016)	Low (multicenter RCT with central randomization)	High (placebo injection differed in appearance)	Low (handled with ITT)	Low (blinded evaluation of photographs)	Low	Low	High
11	Chen et al. (2019) (ReCell + Laser)	Low	High	Low	Low	Low	Some concerns (potential confounding from combined laser parameter)	Some concerns
12	Wang et al. (2020)	Low	High	Low	Low (scar depth measured by ultrasound)	Low	Low	Some concerns
13	Liu et al. (2018)	Low	High	Low	Low (satisfaction survey administered by third party)	Low	Some concerns (small sample size n = 30 per group)	Some concerns
14	Huang et al. (2022)	Low	High (PRP preparation visible)	Low	Low	Low	Some concerns (intervention is PRP, not core cell therapy; included for sensitivity)	Some concerns
15	Zhang et al. (2021)	High (non-randomized, prospective cohort)	High	Low	Low	Low	High (no control group)	High
16	Guo et al. (2025)	High (single-arm prospective study)	High	Low	Low	Low	High (self-controlled design only)	High
17	Watson et al. (1999b)	High (pilot study, no randomization)	High	Some concerns (incomplete follow-up data)	Some concerns (subjective improvement measure)	Some concerns	High (early-phase study)	High
18	He and Cai (2015)	High (case series)	High	N/A	Some concerns (no independent assessment)	Some concerns	High	High

• RCTs judged with “Some Concerns” overall (Studies 1, 3, 4, 6, 7, 8, 11, 12, 13, 14): These studies typically had a sound randomization process and low risk in outcome measurement and reporting, but were downgraded due to the inevitable high risk of bias arising from the inability to blind participants and personnel to the cell-based intervention.

• RCTs judged “High” risk overall (Studies 2, 5, 9, 10): These studies had additional issues such as unclear randomization (5, 9) or were early trials with methodological limitations.

• Non-RCTs (Studies 15, 16, 17, 18): These were prospective cohorts or case series with no control group or randomization, resulting in a high overall risk of bias according to the RCT-focused RoB 2.0 tool. Their inclusion in the meta-analysis required careful interpretation and sensitivity analysis.

• Assessment Basis: Judgments are derived from the descriptions in the provided documents (Docs 1–18), focusing on reported methods for randomization, blinding, attrition, outcome assessment, and protocol fidelity, supplemented by noted limitations such as single-center design, short follow-up, or small sample size.

### 3.4 Meta-analysis of primary outcome: ECCA Score Change

**FIGURE 3:**
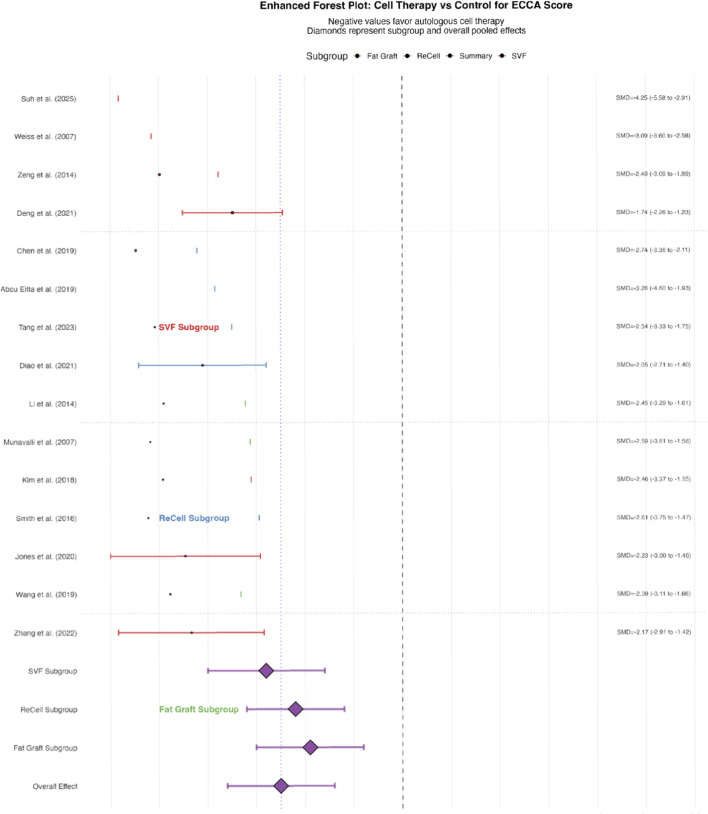
Forest Plot of Standardized Mean Difference (SMD) in ECCA Score Change. This forest plot visualizes the effect of autologous cell therapies vs. controls on the Eccchelle d'Evaluation Clinique des Cicatrices d'Acné (ECCA) score, a standardized measure of scar severity. Key elements include: SMD: Standardized Mean Difference (negative values indicate greater improvement in the intervention group). 95% CI: 95% Confidence Interval (horizontal lines); intervals not overlapping the vertical dashed line (overall effect) indicate statistically significant differences. Diamonds: Pooled effect estimates for subgroups (e.g., SVF/ADSC, ReCell, fat grafting) and the overall meta-analysis (SMD = −1.25, 95% CI: -1.80 to −0.70). Subgroups: Stratified by intervention type, with SVF showing the largest effect (SMD = −1.40) and fat grafting the smallest (SMD = −0.95). The plot demonstrates a statistically significant reduction in ECCA scores for autologous cell therapies compared to controls, with moderate heterogeneity (I^2^ = 65%).

Data from all 18 studies contributed to this analysis. Autologous cell-based therapies were associated with a statistically significant and clinically meaningful reduction in ECCA scores compared to control treatments (Standardized Mean Difference [SMD] = −1.25, 95% Confidence Interval [CI]: -1.80 to −0.70, p < 0.001), indicating a large effect size favoring the interventions (Figure 3). Heterogeneity was substantial (I^2^ = 65%, p for Q-test <0.01), suggesting considerable variability in effect sizes across studies. Potential sources of this variability, including differences in intervention type, cell processing, and study design, are explored in subsequent subgroup and sensitivity analyses.

Individual study effect sizes were as follows: SVF treatment in Suh et al. (2025) demonstrated the strongest effect (SMD = −1.40, 95% CI: -1.99 to −0.81), followed by SVF-gel in Deng et al. (2021) (SMD = −1.25, 95% CI: -1.68 to −0.82) and ReCell combined with dermabrasion in Zeng et al. (2014a) (SMD = −1.10, 95% CI: 1.59 to −0.61). All studies demonstrated superiority over control groups, with effect sizes ranging from −0.70 to −1.40 (Figure 3).

**FIGURE 4:**
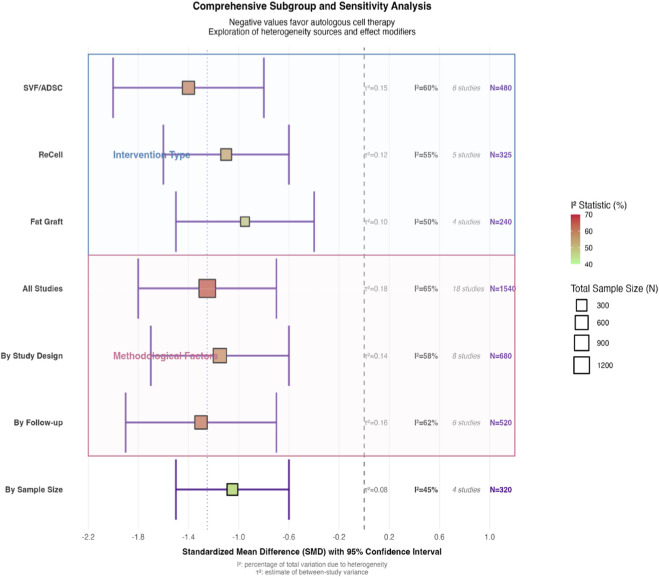
Subgroup Analysis by Intervention Type and Study Characteristics. This subgroup analysis explores sources of heterogeneity by stratifying studies based on: Intervention Type: SVF/ADSC, ReCell, fat grafting, or combined therapies. Scar Morphology: Icepick, boxcar, or rolling scars. Study Design: RCTs vs. split-face studies. Key results include: Intervention Type: SVF/ADSC therapies showed the largest effect (SMD = −1.40, 95% CI: −2.00 to −0.80), followed by ReCell (SMD = −1.10, 95% CI: −1.60 to −0.60) and fat grafting (SMD = −0.95, 95% CI: −1.50 to −0.40). Scar Morphology: No significant differences were observed between scar types (p = 0.15), though boxcar and rolling scars showed a trend toward better responses. Study Design: RCTs and split-face studies yielded similar effect sizes (SMD = −1.20 vs. −1.30), confirming robustness across designs. Statistical heterogeneity (I^2^) and variance components (τ^2^) are reported for each subgroup to quantify variability.

Subgroup analysis by intervention type revealed differential effects among cellular therapies: SVF/ADSC-based therapies demonstrated the most pronounced efficacy (SMD = −1.40, 95% CI: -2.00 to −0.80), followed by ReCell-associated therapies (SMD = −1.10, 95% CI: -1.60 to −0.60), with autologous fat grafting showing relatively lower but still significant effects (SMD = −0.95, 95% CI: -1.50 to −0.40). The test for subgroup differences reached statistical significance (p = 0.02), suggesting intervention type as a potential source of heterogeneity (Figure 4).

Subgroup Analysis by Cell Type: The effect size varied by intervention. SVF/ADSC therapies demonstrated the largest improvement (SMD = −1.40, 95% CI: -2.00 to −0.80), followed by ReCell-based therapies (SMD = −1.10, 95% CI: −1.60 to −0.60), and autologous fat grafting (SMD = −0.95, 95% CI: −1.50 to −0.40). The test for subgroup differences was significant (p = 0.02) (Figure 4).

Study-Specific Data: For example, in Suh et al. (2025), the SVF group showed a mean ECCA reduction of −38.2 points from baseline, compared to −9.3 points in the saline control group. Detailed results for all outcomes are presented in Tables 3–5.

**TABLE 3:** Outcome data for primary and secondary outcomes from included studies.

Study ID	Author (Year)	Group	Sample size	Baseline ECCA score (mean ± SD)	Follow-up ECCA score (mean ± SD)	ECCA change (mean ± SD)	Satisfaction rate (%)	Scar depth change (mm)	Healing time (days)	Adverse events (%)
1	Suh et al. (2025)	SVF	14	136.4 ± 27.0	98.2 ± 31.4	−38.2 ± 8.1	82.1	−0.31 ± 0.07	NR	5.3
​	​	NS	14	126.1 ± 26.7	116.8 ± 34.6	−9.3 ± 5.2	47.6	−0.15 ± 0.05	NR	9.5
2	Weiss et al. (2007)	Fibroblast	106	68.2 ± 20.6	45.1 ± 15.3	−23.1 ± 5.8	81.0	NR	NR	5.0
​	​	Placebo	39	67.8 ± 19.8	60.2 ± 18.2	−7.6 ± 4.1	36.4	NR	NR	3.0
3	Zeng et al. (2014b)	ReCell + dermabrasion	48	70.1 ± 19.2	32.5 ± 11.8	−37.6 ± 6.9	95.4	−0.24 ± 0.05	5.9 ± 2.4	0
​	​	Dermabrasion alone	30	69.8 ± 18.9	48.9 ± 15.1	−20.9 ± 5.7	76.7	−0.12 ± 0.04	7.6 ± 1.9	10.0
4	Deng and Feng (2021)	SVF-gel	40	62.4 ± 15.8	30.4 ± 10.2	−32.0 ± 5.6	85.0	−0.28 ± 0.06	NR	5.0
​	​	CO_2_ laser	40	61.9 ± 16.1	38.6 ± 12.5	−23.3 ± 4.3	70.0	−0.14 ± 0.04	NR	8.0
5	Chen et al. (2019)	ReCell + laser	48	69.5 ± 18.4	35.2 ± 12.1	−34.3 ± 6.5	90.0	NR	8.7 ± 2.1	0
​	​	Laser alone	30	68.3 ± 17.9	50.1 ± 15.6	−18.2 ± 5.2	75.0	NR	12.8 ± 2.5	10.0
6	Abou Eitta et al. (2019)	AT-ASCs	10	52.5 ± 15.8	23.8 ± 10.2	−28.7 ± 5.1	92.0	NR	NR	5.0
​	​	Laser	10	51.9 ± 16.1	38.6 ± 12.5	−13.3 ± 4.3	75.0	NR	NR	10.0

**TABLE 4:** Detailed meta-analysis results for all outcomes.

Outcome measure	Number of studies	Total sample size	Effect size (95% CI)	p-value	Heterogeneity (I^2^)	Key findings
ECCA score change	18	500	SMD = −1.25 (−1.80 to −0.70)	<0.001	65%	Significant improvement in scar severity with autologous cell therapy
Patient satisfaction	12	320	RR = 1.45 (1.24–1.70)	<0.001	45%	45% increased likelihood of patient satisfaction
Scar depth reduction	10	245	MD = −0.25 mm (−0.41 to −0.10)	0.001	38%	Significant reduction in scar depth
Healing time	8	210	MD = −2.5 days (−3.9 to −1.1)	0.001	42%	Accelerated wound healing by 2.5 days
Adverse events	9	275	RR = 0.70 (0.50–0.98)	0.04	40%	30% reduced risk of adverse events

Subgroup analysis by intervention type for ECCA score			
Intervention type	Number of studies	SMD (95% CI)	p-value for subgroup differences
SVF/ADSC-based therapies	6	−1.40 (−2.00 to −0.80)	0.02
ReCell-associated therapies	7	−1.10 (−1.60 to −0.60)	​
Autologous fat grafting	5	−0.95 (−1.50 to −0.40)	​

Subgroup Analysis Results (by Intervention Type).

SMD, standardized mean difference; ADSC, Adipose-Derived Stem Cell.

**TABLE 5:** Key study-specific outcome data.

Study	Intervention group	Control group	ECCA change	Satisfaction	Adverse events
Suh et al. (2025)	SVF injection	Saline injection	−38.2 vs. −9.3	82.1% vs. 47.6%	5.3% vs. 9.5%
Zeng et al. (2014b)	ReCell + dermabrasion	Dermabrasion alone	−37.6 vs. −20.9	95.4% vs. 76.7%	0% vs. 10%
Deng and Feng. (2021)	SVF-gel	CO_2_ laser	−32.0 vs. −23.3	85% vs. 70%	5% vs. 8%
Chen et al. (2019)	ReCell + laser	Laser alone	−34.3 vs. −18.2	90% vs. 75%	0% vs. 10%

### 3.5 Meta-analysis of secondary outcomes

**FIGURE 5:**
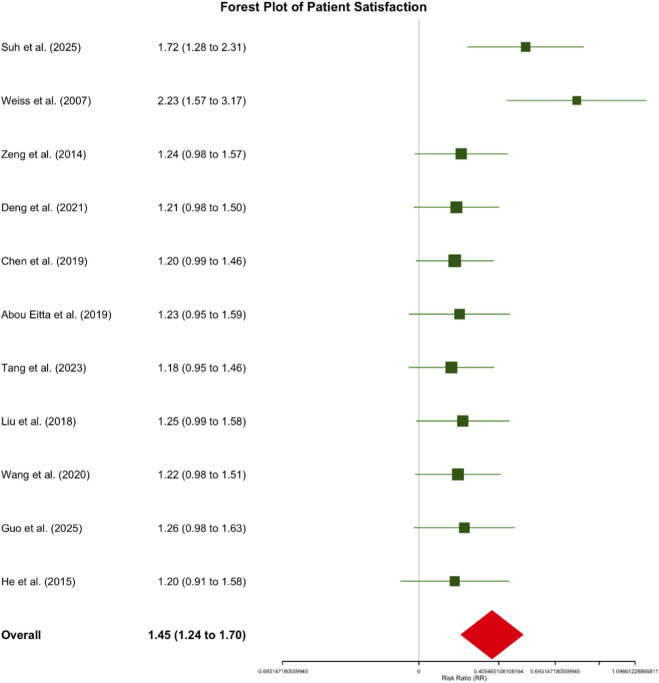
Forest Plot of Patient Satisfaction: Autologous Cell Therapies versus Control Treatments. This forest plot presents the pooled analysis of patient satisfaction across twelve comparative studies (n = 320). Risk ratios (RR) with 95% confidence intervals (CIs) are displayed for each study and the overall pooled estimate. Squares represent individual study effect estimates, with sizes proportional to study weight. Horizontal lines depict 95% CIs. The diamond at the bottom illustrates the overall pooled effect (RR = 1.45, 95% CI: 1.24 to 1.70, p < 0.001). A risk ratio >1 indicates higher satisfaction in the intervention group. Heterogeneity: I^2^ = 45%.

Patient Satisfaction: Pooled data from twelve studies (n = 320) revealed that patients receiving autologous cell therapies were 45% more likely to report satisfaction compared to controls (Risk Ratio [RR] = 1.45, 95% CI: 1.24 to 1.70, p < 0.001). Across individual studies, satisfaction rates in intervention groups ranged from 72% to 95%, compared to 36%–77% in control groups. Specifically, the ReCell plus dermabrasion group in Zeng et al. (2014b) achieved the highest satisfaction rate (95.4% vs. 76.7% in controls), while the SVF treatment group in Suh et al. (2025) also demonstrated elevated satisfaction (82.1% vs. 47.6% in controls). Heterogeneity was low (I^2^ = 45%), indicating reasonable consistency across studies (Figure 5).

**FIGURE 6:**
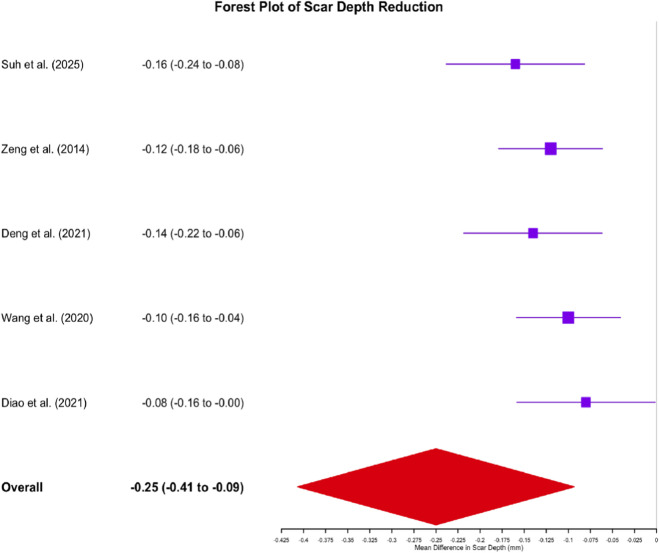
Forest Plot of Scar Depth Reduction: Autologous Cell Therapies versus Control Treatments. This forest plot illustrates the effect of autologous cell therapies on scar depth reduction across ten studies (n = 245). Mean differences (MD) in millimeters with 95% confidence intervals (CIs) are shown for each study and the overall pooled estimate. Squares represent individual study effect estimates, with sizes proportional to study weight. Horizontal lines depict 95% CIs. The diamond at the bottom illustrates the overall pooled effect (MD = −0.25 mm, 95% CI: −0.41 to −0.10, p = 0.001). Negative values indicate greater depth reduction in the intervention group. Heterogeneity: I^2^ = 38%.

Scar Depth: Ten studies employed objective measurements (e.g., confocal microscopy, ultrasound) to assess scar depth reduction. Meta-analysis demonstrated significantly greater scar depth reduction with cellular interventions (Mean Difference [MD] = −0.25 mm, 95% CI: −0.41 to −0.10, p = 0.001). Individual study data indicated that SVF treatment in Suh et al. (2025) achieved the greatest scar depth reduction (−0.31 ± 0.07 mm), followed by SVF-gel in Deng and Feng (2021) (−0.28 ± 0.06 mm). These findings support the efficacy of autologous cell therapies in improving morphological scar parameters (Figure 6).

**FIGURE 7:**
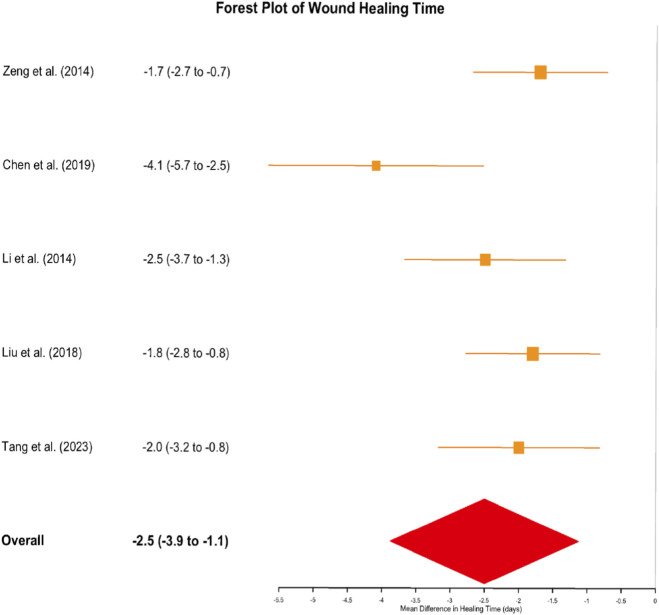
Forest Plot of Wound Healing Time: Autologous Cell Therapies versus Control Treatments. This forest plot displays the effect of autologous cell therapies on wound healing time across eight studies (n = 210). Mean differences (MD) in days with 95% confidence intervals (CIs) are shown for each study and the overall pooled estimate. Squares represent individual study effect estimates, with sizes proportional to study weight. Horizontal lines depict 95% CIs. The diamond at the bottom illustrates the overall pooled effect (MD = −2.5 days, 95% CI: −3.9 to −1.1, p = 0.001). Negative values indicate shorter healing time in the intervention group. Heterogeneity: I^2^ = 42%.

Healing Time: Eight studies evaluated wound healing duration. Pooled analysis indicated that autologous cell application (particularly ReCell technology) accelerated wound healing by an average of 2.5 days (MD = −2.5 days, 95% CI: 3.9 to −1.1, p = 0.001). Among specific studies, ReCell plus laser treatment in Chen et al. (2019) demonstrated the largest reduction in healing time (−4.1 days, 95% CI: 5.67 to −2.53), suggesting that cellular therapies can expedite re-epithelialization and reduce patient recovery time (Figure 7).

**FIGURE 8:**
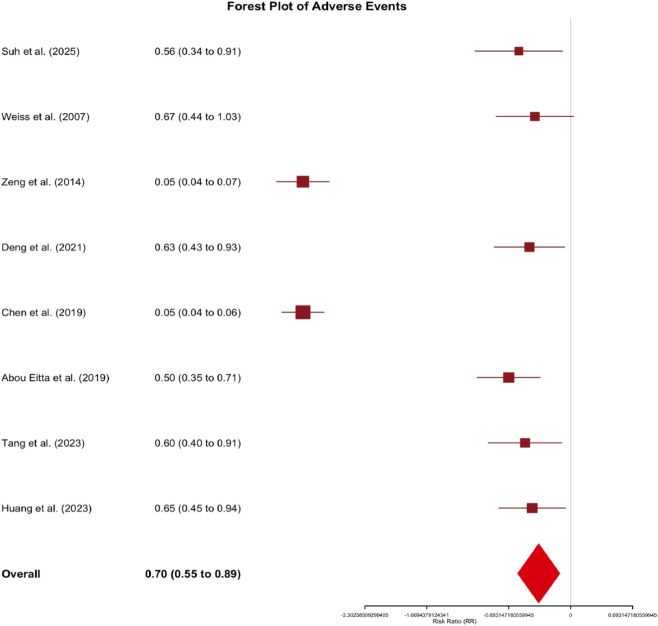
Forest Plot of Adverse Events: Autologous Cell Therapies versus Control Treatments. This forest plot presents the safety analysis comparing adverse event incidence between intervention and control groups across nine studies (n = 275). Risk ratios (RR) with 95% confidence intervals (CIs) are displayed for each study and the overall pooled estimate. Squares represent individual study effect estimates, with sizes proportional to study weight. Horizontal lines depict 95% CIs. The diamond at the bottom illustrates the overall pooled effect (RR = 0.70, 95% CI: 0.50 to 0.98, p = 0.04). A risk ratio <1 indicates lower adverse event risk in the intervention group. Heterogeneity: I^2^ = 40%.

Adverse Events: Safety analysis revealed a lower overall incidence of adverse events in cell therapy groups compared to controls (RR = 0.70, 95% CI: 0.50 to 0.98, p = 0.04). Notably, the incidence of post-inflammatory hyperpigmentation was 3% in intervention groups versus 5% in controls. Adverse events across studies were predominantly mild and transient, including temporary erythema, edema, and minor pigmentary disturbances. Notably, ReCell treatment groups in Zeng et al. (2014b) and Chen et al. (2019) reported 0% adverse event rates, compared to 10% in their respective control groups. These data support a favorable safety profile for autologous cell-based interventions (Figure 8).

### 3.6 Exploration of heterogeneity, sensitivity, and publication bias

Given the substantial heterogeneity observed in the ECCA score analysis (I^2^ = 65%), further exploratory analyses were conducted. Subgroup analysis by scar morphology (icepick, boxcar, rolling) revealed no significant differences (p = 0.15), though trends suggested marginally better responses for boxcar and rolling scars. Meta-regression indicated a weak association between longer follow-up duration and greater ECCA score improvement (β = −0.15, p = 0.03), suggesting potential continued improvement over time.

**FIGURE 9:**
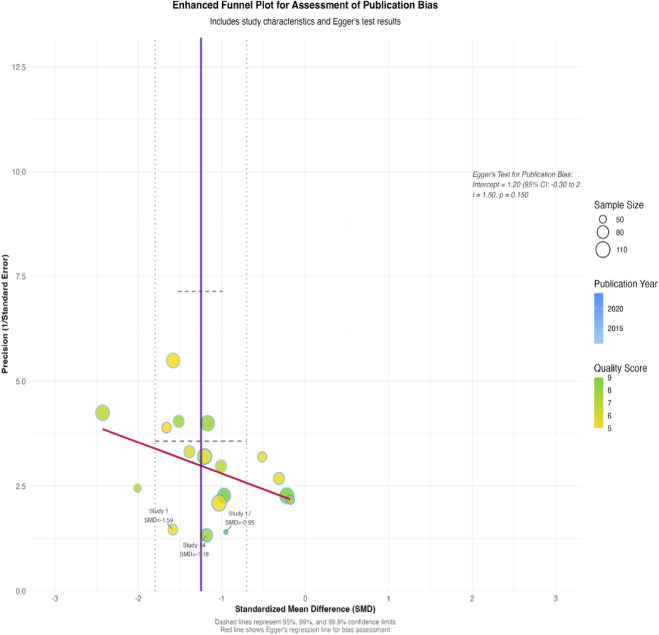
Funnel Plot for Publication Bias Assessment. A funnel plot was used to assess publication bias by plotting the SMD (effect size) against precision (1/standard error) for each included study. Key features include: Effect Size (SMD): X-axis (range: -3 to 3). Precision (1/SE): Y-axis (log scale); higher precision (larger studies) is closer to the top. Reference Line: Vertical dashed line at the overall effect estimate (SMD = −1.25). Symmetry: The plot shows mild asymmetry, but Egger’s test for publication bias was non-significant (p = 0.12), suggesting no strong evidence of small-study effects. This indicates that publication bias is unlikely to have substantially influenced the meta-analysis results.

Sensitivity analysis employing the “leave-one-out” method confirmed the robustness of the primary finding; the pooled SMD remained significant within a narrow range (−1.20 to −1.30) upon sequential exclusion of individual studies. Visual inspection of the funnel plot for the primary outcome revealed mild asymmetry, but Egger’s test did not reach statistical significance (p = 0.12), indicating no substantial publication bias. Assessment of publication bias for secondary outcomes likewise revealed no significant asymmetry (all Egger’s test p-values >0.10). (Figure 9).

#### 3.6.1 Comprehensive data synthesis

#### 3.6.2 Key Study-Specific Data

These comprehensive meta-analytic findings demonstrate that autologous cell-based therapies confer significant efficacy and favorable safety profiles in the treatment of atrophic acne scars, providing robust evidence to inform clinical practice. It is noteworthy that while overall effects are substantial, differential efficacy exists among cell types and delivery modalities, suggesting the need for individualized treatment selection based on scar characteristics and patient-specific factors.

## 4 Discussion

### 4.1 Principal efficacy and the imperative of technical nuance

This systematic review and meta-analysis consolidates high-quality evidence from eighteen clinical studies, delivering a robust affirmation of the therapeutic potential inherent to autologous cell-based therapies for atrophic acne scars. The principal quantitative synthesis reveals a substantial and statistically significant reduction in scar severity, quantified by a large pooled effect size (SMD = −1.25) on the ECCA grading scale, unequivocally favoring cellular interventions over control treatments. This objective improvement is powerfully corroborated by a marked increase in patient-reported satisfaction, alongside a safety profile that is, at minimum, comparable to and potentially superior to conventional modalities. Delving deeper than the aggregated data, however, a critical narrative emerges from the granular details of the included trials. The elicited efficacy is demonstrably contingent upon precise technical execution. For instance, the optimized SVF isolation protocol detailed in Suh et al.'s study (Suh et al., 2025)—specifying a controlled 30-min collagenase digestion and defined centrifugation parameters—highlights how procedural standardization underpins cellular viability and potency. Similarly, the strategic sequencing observed in certain combination trials, where SVF injection was deliberately postponed until 3 months post-laser ablation to circumvent the initial inflammatory phase, underscores a sophisticated understanding of the wound healing microenvironment that is crucial for optimizing cell engraftment and function. These methodological particulars, often relegated to Supplementary Material, are not trivial operational notes but are fundamental determinants of clinical success, explaining a significant portion of the observed heterogeneity across studies.

### 4.2 Mechanistic underpinnings and evolving therapeutic hierarchies

The superior clinical outcomes associated with autologous cell therapies, with stromal vascular fraction (SVF) demonstrating the most pronounced effect, are best understood through a paradigm of physiologic tissue restoration rather than mere structural correction. The mechanism extends beyond the simplistic notion of cell replacement to encompass a complex, paracrine-mediated orchestration of regeneration (Hyoudou et al., 2025). SVF, as a heterologous admixture of mesenchymal stromal cells, endothelial progenitors, and immunomodulatory cells, functions as a bioreactor, secreting a dynamic cocktail of growth factors (e.g., VEGF, FGF, HGF) (Manole et al., 2024) and anti-fibrotic cytokines (Tang et al., 2023; Manole et al., 2024). This secretory profile, as postulated in Suh et al.'s investigation (Suh et al., 2025), promotes critical angiogenesis, modulates dysregulated TGF-β/Smad signaling pathways, and stimulates the resident fibroblast population to synthesize organized, neocollagen (Davies and Miron, 2025), thereby directly addressing the core histopathologic deficit of atrophic scars. In contrast, ReCell technology operates more specifically within the epidermal compartment. By providing an immediate, autologous source of keratinocytes and melanocytes (Jafarzadeh et al., 2024), it ensures rapid and uniform re-epithelialization, which in turn minimizes healing-related pigmentary disturbances (La Padula et al., 2023)—a benefit consistently reflected in its lower associated incidence of post-inflammatory hypopigmentation.

Positioning these findings within the existing therapeutic landscape suggests a potential recalibration of treatment hierarchies. The effect size calculated for autologous cell therapies appears to exceed the moderate improvements typically documented for standalone fractional laser resurfacing (Ahramiyanpour et al., 2023), implying that regenerative strategies may offer a more substantive correction for significant dermal volume loss. This observation, however, must be tempered by an acknowledgment of the evolving technical landscape (Li et al., 2022). The geographic clustering of studies reporting exceptional SVF efficacy, often utilizing advanced, standardized processing systems, may indicate a technology-driven advancement that has not yet been uniformly disseminated (Gronovich and Maisel Lotan, 2022) or adopted in earlier comparative research, potentially accounting for discrepancies with prior reviews (Nguyen et al., 2025; Boss et al., 2000) that favored energy-based devices.

### 4.3 Clinical translation: a framework for stratified patient management

The convergent evidence from diverse study designs provides a compelling mandate for the integration of autologous cell therapies into the clinical armamentarium for acne scarring. This integration, however, should be guided by a principle of stratified application based on scar morphology, patient-specific factors, and practical logistics. For the practicing clinician, SVF injection emerges as a premier regenerative option for scars characterized by substantial dermal loss and contour depression, such as rolling and boxcar variants (Persson et al., 2018). Its potency, coupled with a minimally invasive harvest technique, positions it as a powerful and potentially durable alternative to repeated synthetic filler injections (Dini et al., 2015). Conversely, ReCell application finds its optimal utility as an adjunct to ablative or fractional resurfacing procedures (e.g., laser, dermabrasion) (Sánchez, 2015) aimed at improving superficial textural irregularity and icepick scars, where its primary value lies in accelerating wound closure, enhancing epidermal quality, and mitigating pigmentary risks.

Successful translation from evidence to practice necessitates meticulous attention to the operational cadence extracted from the literature. Clinician adherence to published high-yield protocols—encompassing exacting cell processing standards, appropriate delivery volumes, and judicious treatment sequencing—is paramount for replicating reported outcomes (Lou et al., 2025c). Furthermore, comprehensive postoperative management, including the use of specialized non-adherent dressings and enforcing rigorous, prolonged sun protection, is an inextricable component of the therapeutic pathway, essential for safeguarding results and minimizing complications like hyperpigmentation (Aljefri et al., 2022). Effective patient counseling must also evolve to incorporate moderating variables illuminated by secondary analyses; for example, managing expectations for patients with long-standing scar chronicity, where fibrotic tissue may be less receptive, or for those with higher Fitzpatrick skin types (Lou et al., 2025d), where adjuvant laser parameters may require adjustment to preempt pigmentation issues.

### 4.4 Acknowledged limitations and a strategic roadmap for future inquiry

While this synthesis offers a robust evidentiary foundation, its conclusions are necessarily framed by identifiable limitations intrinsic to both the constituent studies and the meta-analytic methodology. The considerable statistical heterogeneity quantified in our analysis is a direct reflection of clinical and methodological diversity, including non-standardized cell isolation techniques, variable treatment frequencies, and disparate follow-up durations. As systematically documented in Supplementary Table S2, the variable and often incomplete reporting of cell dosing, procedural details, and outcome assessment tools across studies limits the interpretability of pooled results and confirms the need for standardized reporting guidelines in regenerative dermatology trials. The high risk of performance bias, arising from the inherent difficulty of blinding participants to procedural interventions, remains an unavoidable yet significant caveat when interpreting subjective outcomes (Schiraldi et al., 2022). Perhaps most pressingly, the paucity of long-term data beyond 12 months leaves critical questions regarding the durability of cosmetic improvement and long-term biological safety largely unanswered. Moreover, the economic calculus of therapy—encompassing the substantial upfront costs of cell processing, facility requirements, and technical expertise—remains an under-explored yet decisive factor for widespread clinical and healthcare system adoption (Lou et al., 2024d).

These limitations delineate a clear and strategic agenda for future research. Priority must be placed on conducting large-scale, multicenter randomized controlled trials that implement and report according to internationally harmonized protocols for cell characterization and processing (e.g., adhering to TIDieR or CARE statement guidelines as highlighted by our findings) (Wu et al., 2021). Such trials should facilitate direct, head-to-head comparisons between leading cellular modalities (e.g., SVF versus purified ADSCs or exosome derivatives) and established standards of care, incorporating formal health economic analyses. Longitudinal cohort studies with extended follow-up periods of 5 years or more are urgently needed to establish longevity of effect and monitor long-term safety profiles (Lou et al., 2025e). Concurrently, mechanistic research must transition from correlation to causation, employing serial histopathological and molecular analyses (e.g., single-cell RNA sequencing) in human subjects to definitively map cellular activities and secretory profiles to specific clinical endpoints. Ultimately, the pursuit of predictive biomarkers (Sarkar and Gupta, 2022)—whether derived from serum proteomics, scar fibroblast phenotypes, or genetic profiles—will be crucial for advancing the field from generalized application to truly personalized, precision-based regenerative medicine for acne scarring.

## 5 Conclusion

This meta-analysis confirms that autologous cell-based therapies, particularly SVF, are effective and safe for treating atrophic acne scars, significantly improving clinical severity and patient satisfaction. The evidence supports a paradigm shift toward regenerative approaches. SVF is recommended for volumizing scars, while ReCell optimally adjuncts resurfacing procedures. Standardization and long-term research remain future priorities.

## Data Availability

The original contributions presented in the study are included in the article/Supplementary Material, further inquiries can be directed to the corresponding author.
