# Supplementary material for: Efficacy and safety of autologous cell-based therapies for atrophic acne scar treatment: an updated systematic review and meta-analysis with in-depth methodological and clinical insights
Source: Front Cell Dev Biol. 2026 Mar 11;14:1773607. doi: 10.3389/fcell.2026.1773607 (PMC13013385; doi:10.3389/fcell.2026.1773607)
Supplement: Supplementary file 1 [file Table1.docx]

| **Supplementary Table S1: Complete Search Strategies for All Electronic Databases** | | | |
| --- | --- | --- | --- |
| **Database** | **Platform / Interface** | **Date of Search** | **Search Strategy** |
| **PubMed** | <https://pubmed.ncbi.nlm.nih.gov/> | Dec 22, 2025 | ("acne scar*"[Title/Abstract] OR "atrophic acne"[Title/Abstract] OR "acne cicatrix"[MeSH Terms]) AND ("autologous cell therapy"[Title/Abstract] OR "stromal vascular fraction"[Title/Abstract] OR "SVF"[Title/Abstract] OR "ReCell"[Title/Abstract] OR "autologous skin cell suspension"[Title/Abstract] OR "adipose-derived stem cells"[Title/Abstract] OR "fat grafting"[MeSH Terms] OR "transplantation, autologous"[MeSH Terms]) AND ("randomized controlled trial"[Publication Type] OR "controlled clinical trial"[Publication Type] OR "randomized"[Title/Abstract] OR "randomly"[Title/Abstract] OR "trial"[Title/Abstract] OR "split-face"[Title/Abstract]) NOT (animals[MeSH Terms] NOT humans[MeSH Terms]) |
| **Embase** | OvidSP | Dec 22, 2025 | 1. exp acne scar/ 2. (acne scar* or atrophic acne or acne cicatrix).tw,kw. 3. 1 or 2 4. exp autologous cell therapy/ 5. exp stromal vascular fraction/ 6. exp adipose derived stem cell/ 7. exp fat grafting/ 8. (autologous cell therapy or stromal vascular fraction or SVF or ReCell or autologous skin cell suspension or adipose-derived stem cell* or fat graft*).tw,kw. 9. 4 or 5 or 6 or 7 or 8 10. exp randomized controlled trial/ 11. (random* or placebo or split-face or split face).tw,kw. 12. 10 or 11 13. 3 and 9 and 12 14. limit 13 to human |
| **Cochrane Central Register of Controlled Trials (CENTRAL)** | The Cochrane Library (Wiley) | Dec 22, 2025 | #1 MeSH descriptor: [Acne Vulgaris] explode all trees #2 (acne NEXT scar*):ti,ab,kw OR "atrophic acne":ti,ab,kw OR "acne cicatrix":ti,ab,kw #3 #1 OR #2 #4 MeSH descriptor: [Transplantation, Autologous] explode all trees #5 MeSH descriptor: [Adipose Tissue] explode all trees #6 (autologous cell therapy):ti,ab,kw OR (stromal vascular fraction):ti,ab,kw OR SVF:ti,ab,kw OR ReCell:ti,ab,kw OR (autologous skin cell suspension):ti,ab,kw OR (adipose-derived stem cell*):ti,ab,kw OR (fat graft*):ti,ab,kw #7 #4 OR #5 OR #6 #8 #3 AND #7 Publication Year from 1900 to 2025 |
| **Web of Science Core Collection** | Clarivate Analytics | Dec 22, 2025 | TS=("acne scar*" OR "atrophic acne" OR "acne cicatrix") AND TS=("autologous cell therapy" OR "stromal vascular fraction" OR SVF OR ReCell OR "autologous skin cell suspension" OR "adipose-derived stem cell*" OR "fat graft*") AND TS=(random* OR RCT OR "controlled trial" OR "split-face" OR "split face") Refined by: [excluding] DOCUMENT TYPES: ( MEETING ABSTRACT OR PROCEEDINGS PAPER OR BOOK CHAPTER ) AND LANGUAGES: ( ENGLISH OR CHINESE ) Timespan: All years. |
| **China National Knowledge Infrastructure (CNKI)** | <https://www.cnki.net/> | Dec 22, 2025 | **主题**=(痤疮瘢痕 OR 凹陷性痤疮 OR 痘坑) AND **主题**=(自体细胞治疗 OR 基质血管组分 OR SVF OR 瑞细胞 OR 自体皮肤细胞悬液 OR 脂肪来源干细胞 OR 脂肪移植) AND **主题**=(随机 OR 对照 OR 自身对照) **时间范围**: 建库至 2025-12-22 **文献来源**: 学术期刊, 博硕士论文, 会议论文 |
| **Wanfang Data** | <https://www.wanfangdata.com.cn/> | Dec 22, 2025 | **题名或关键词**:(痤疮瘢痕 + 凹陷性痤疮 + 痘坑) * **题名或关键词**:(自体细胞 + 基质血管组分 + SVF + 瑞细胞 + 脂肪干细胞 + 脂肪移植) * **题名或关键词**:(随机 + 对照 + 临床试验) **时间范围**: 所有年份 **文献类型**: 期刊论文, 学位论文, 会议论文 |
| **VIP Database** | <http://www.cqvip.com/> | Dec 22, 2025 | U=(痤疮瘢痕 + 凹陷性痤疮) * U=(自体细胞治疗 + 基质血管片段 + SVF + ReCell + 脂肪移植) * U=(随机 + 对照) |
| **Notes:**   1. **Search Filters:** No methodological filters were applied to limit study design in the primary search strings, beyond the terms related to randomization/trials included in the strategy. Language filters were not applied at the database level. 2. **Adaptation:** The strategy was conceptually adapted across databases using appropriate subject headings (MeSH, EMTREE) and syntax for each platform. 3. **Translation:** For Chinese databases (CNKI, Wanfang, VIP), search terms were professionally translated to capture the relevant literature. | | | |
