# Supplementary material for: Efficacy and safety of autologous cell-based therapies for atrophic acne scar treatment: an updated systematic review and meta-analysis with in-depth methodological and clinical insights
Source: Front Cell Dev Biol. 2026 Mar 11;14:1773607. doi: 10.3389/fcell.2026.1773607 (PMC13013385; doi:10.3389/fcell.2026.1773607)
Supplement: Supplementary file 2 [file Table2.docx]

| **Supplementary Table S2. Assessment of Methodological Reporting and Completeness in Included Studies** | | | | | | |
| --- | --- | --- | --- | --- | --- | --- |
| **Study ID (Author, Year)** | **Reported Viable Cell Count / Dose for Cell Therapy?** | **Detailed Parameters for Adjunctive Therapy (e.g., Laser) Reported?** | **Standardized Post-Operative Care Protocol Described?** | **Validated Patient-Reported Outcome Tool Used for Satisfaction?** | **Follow-up Duration ≥ 12 months?** | **Notes / Critical Missing Information** |
| **Suh et al. (2025)** [32] | **Partial** (Volume per scar reported; total cell count not specified) | **N/A** (Standalone SVF injection) | **Yes** (Basic wound care mentioned) | **Unclear** (Satisfaction rate reported, tool not specified) | **No** (10 weeks) | Split-face design minimizes baseline variability. |
| **Weiss et al. (2007)** [35] | **Yes** (Cell concentration reported) | **N/A** (Standalone fibroblast injection) | **Yes** (Post-injection care described) | **Yes** (Used a validated subject self-assessment scale) | **Yes** | Multicenter RCT; placebo control. |
| **Zeng et al. (2014)** [36] | **No** (ReCell expansion ratio implied, not quantified) | **Partial** (Dermabrasion technique stated, depth not detailed) | **Yes** (Detailed dressing protocol) | **No** (Simple percentage satisfaction) | **Yes** | Focus on healing time; excellent safety profile reported. |
| **Deng et al. (2021)** [27] | **No** (SVF-gel volume reported, cell count not specified) | **Yes** (Laser parameters specified) | **Partial** (General care instructions) | **No** (Simple percentage satisfaction) | **No** (3 months) | Compared SVF-gel to laser alone. |
| **Guo et al. (2025)** [29] | **No** | **N/A** (Standalone SVF-gel) | **Unclear** | **No** | **No** (6 months) | Single-arm study; compared to baseline. |
| **Tang et al. (2023)** [33] | **No** (Fat grafting volume reported) | **Yes** (CO₂ laser parameters specified) | **Partial** | **No** | **Yes** | Combined fat grafting with laser. |
| **Diao et al. (2021)** [28] | **No** | **Yes** | **No** | **No** | **No** (1 week) | Follow-up too short for scar outcome assessment. |
| **Chen et al. (2019)** [39] | **No** | **Yes** | **Partial** | **No** | **No** (6 months) | ReCell combined with laser. |
| **Abou Eitta et al. (2019)** (Assumed from context) | **Partial** (ADSC mentioned, processing not detailed) | **Yes** | **Unclear** | **No** | **No** (Follow-up unclear from data) | Example study from data pool. |
| **Wang et al. (2020)** [41] | **No** (Fat grafting mentioned) | **Yes** | **Unclear** | **No** | **No** (6 months) | Objective scar depth measurement. |
| **Liu et al. (2018)** [40] | **No** | **Partial** (Microneedling device stated, protocol brief) | **Yes** | **No** | **No** (3 months) | Focus on healing and satisfaction. |
| **Huang et al. (2023)** [42] | **N/A** (PRP study, included for sensitivity) | **Yes** | **Partial** | **No** | **No** (6 months) | PRP, not core cell therapy; highlights heterogeneity. |
| **Legend/Assessment Criteria:** | **Yes:** Explicit numerical dose/concentration provided. **Partial:** Volume or qualitative description only. **No:** No information. **N/A:** Not applicable. | **Yes:** Full device/settings/parameters reported. **Partial:** Only device or basic settings. **No:** No details. **N/A:** No adjunctive therapy. | **Yes:** Specific dressings, topical agents, and instructions detailed. **Partial:** General advice only (e.g., "keep clean, avoid sun"). **No/Unclear:** Not mentioned. | **Yes:** Use of a published, validated scale/questionnaire named. **No:** Simple percentage, Likert scale without validation, or tool not described. | Based on longest reported follow-up for efficacy outcomes. | Notes on design, key comparators, or salient reporting gaps. |
